# Filamin A modulates platelet function

**DOI:** 10.18632/aging.101635

**Published:** 2018-11-06

**Authors:** Jose J. Lopez, Isaac Jardin, Juan A. Rosado

**Affiliations:** 1 Department of Physiology, Cellular Physiology Research Group, Institute of Molecular Pathology Biomarkers, University of Extremadura, 10003 Caceres, Spain

**Keywords:** filamin A, STIM1, platelet function, calcium entry

Platelets or thrombocytes are anucleated cells (or cell fragments) that play a key role in hemostasis when the vessel wall is damaged, either by their participation in the activation of the coagulation cascades or, more directly, by their ability to adhere to subendothelial surfaces and form aggregates. The number of circulating platelets as well as the correct aggregability is essential for an appropriate hemostasis. In addition to their primary function, platelets also play a relevant role in a diversity of events, such as chronic and allergic inflammations through the exocytosis of platelet granules. Platelet function, either adhesion, aggregation or secretion, is tightly regulated by a number of cellular mechanisms, among which the mobilization of intracellular free Ca^2+^ stands out.

Platelet agonists increase cytosolic free-Ca^2+^ concentration by the release of Ca^2+^ from intracellular Ca^2+^ stores, mostly the dense tubular system and acidic lysosomal-like organelles, and the influx of extracellular Ca^2+^ through plasma membrane channels. A major mechanism for agonist-induced Ca^2+^ influx in platelets is store-operated or capacitative Ca^2+^ entry [1]. Store-operated Ca^2+^ entry (SOCE) is a mechanism for Ca^2+^ influx whereby the discharge of intracellular Ca^2+^ stores secondarily activates Ca^2+^ influx across Ca^2+^-permeable channels. A key molecular element of SOCE is the endoplasmic reticulum Ca^2+^ sensor, STIM1 [2], or its homolog STIM2. Current evidence indicates that Ca^2+^ store depletion results in the activation of two types of STIM1-regulated channels, named the Ca^2+^ release-activated Ca^2+^ (CRAC) channels and the store-operated Ca^2+^ (SOC) channels. CRAC channels are highly specific for Ca^2+^ and are composed of Orai1 subunits, while SOC channels are cation permeable channels that consists of still uncharacterized heteromeric complexes of Orai1 and TRPC1 subunits [3]. The existence of *I*_CRAC_ currents in platelets is uncertain but we have reported a functional role for the STIM1-Orai1-TRPC1 ternary complex in intracellular Ca^2+^ homeostasis in these cells [4].

SOCE in platelets requires a profound remodeling of the actin cytoskeleton, which acts as a negative clamp to prevent spontaneous SOCE activation but also is necessary to support the mechanism of Ca^2+^ entry. According to the role of the membrane cytoskeleton as a clamp for constitutive SOCE, we have previously reported that cofilin, an actin-binding protein, is involved in rapid actin filament depolymerization induced by physiological agonists that occurs in a subsecond time scale and, therefore, prior to the detection of SOCE [5]. To illustrate the role of the actin cytoskeleton in the support and modulation of SOCE, we have recently found that filamin A, a 2647-amino acid–long actin-crosslinking protein, plays a relevant role in STIM1 function [6]. Filamin A has been reported to be mainly located in the membrane cytoskeleton where it regulates the interaction between the actin cytoskeleton and membrane receptors. Therefore, filamin A might be a good candidate to regulate the function of plasma membrane ion channels such as those conducting SOCE. Our results indicate that filamin A is phosphorylated at Ser^2152^ by protein kinase A upon Ca^2+^ store depletion and in a Ca^2+^-dependent manner, a post-translational modification that is essential for the interaction of filamin A with STIM1 [6]. Impairment of filamin A Ser^2152^ phosphorylation or attenuation of filamin A expression using RNAi impairs its interaction with STIM1, which results in a significant increase in thapsigargin-induced Ca^2+^ entry, thus indicating that filamin A is a negative modulator of SOCE. In an attempt to ascertain the mechanism underlying the regulatory role of filamin A we found that filamin A knockdown results in an enhancement of the STIM1 interaction with its partner, Orai1, which strongly suggests that filamin A participates in fine-tuning the STIM1-Orai1 interaction in order to generate physiologically adequate Ca^2+^ signals. In the absence of filamin A, an exacerbated STIM1-Orai1 interaction occurs in response to receptor stimulation, leading to aberrant Ca^2+^ influx that might result in abnormally enhanced platelet responses. In fact, our results indicate that impairment of the interaction of filamin A with STIM1 induced by filamin A knockdown results in enhanced platelet aggregation in response to thrombin [6]. These findings provide a further explanation to a recent study reporting that a patient carrying a stop codon mutation in the *FLNA* gene, resulting in a 100-amino acid-long filamin A C-terminal extension, exhibits upregulated platelet aggregation and secretion in response to agonists. This filamin A mutation has been shown to lead to increased α_IIb_β_3_ activation and the enhanced platelet function [7]. Further studies have revealed that a mice model with deficient filamin A expression exhibits platelet hyperaggregability and associated thrombocytopenia due to platelet loss as a result of the enhanced platelet function [8].

Summarizing, filamin A plays a relevant role in fine-tuning the platelet function in response to physiological agonists. The regulation of STIM1-Orai1 interaction, and, therefore, the extent of SOCE to mediate physiologically relevant Ca^2+^ signals, is among the mechanisms underlying the modulatory role of filamin A in platelet function. This study raises several questions in the context of platelet function and the development of hemostatic and cardiovascular disorders. Among the most closely related are the possible involvement of filamin A in uncharacterized thrombotic or bleeding disorders as well as the possible regulation of filamin A expression associated with age.
